# Dynamic Behavior of Rotation Transmission Nano-System in Helium Environment: A Molecular Dynamics Study

**DOI:** 10.3390/molecules26175199

**Published:** 2021-08-27

**Authors:** Pan Zheng, Wugui Jiang, Qinghua Qin, Duosheng Li

**Affiliations:** 1 School of Aeronautical Manufacturing Engineering, Nanchang Hangkong University, Nanchang 330063, China; 15079133743@163.com; 2 Department of Engineering, Shenzhen MSU-BIT University, Shenzhen 518172, China; qinghua.qin@smbu.edu.cn; 3 School of Material Science and Engineering, Nanchang Hangkong University, Nanchang 330063, China; ldsnuaa@nuaa.edu.cn

**Keywords:** rotation transmission nano-system, carbon nanotube, boron nitride nanotube, helium gas, molecular dynamics

## Abstract

The molecular dynamics (MD) method is used to investigate the influence of the shielding gas on the dynamic behavior of the heterogeneous rotation transmission nano-system (RTS) built on carbon nanotubes (CNTs) and boron nitride nanotube (BNNT) in a helium environment. In the heterogeneous RTS, the inner CNT acts as a rotor, the middle BNNT serves as a motor, and the outer CNT functions as a stator. The rotor will be actuated to rotate by the motor due to the interlayer van der Waals effects and the end effects. The MD simulation results show that, when the gas density is lower than a critical range, a stable signal of the rotor will arise on the output and the rotation transmission ratio (RRT) of RTS can reach 1.0, but as the gas density is higher than the critical range, the output signal of the rotor cannot be stable due to the sharp drop of the RRT caused by the large friction between helium and the RTS. The greater the motor input signal of RTS, the lower the critical working helium density range. The results also show that the system temperature and gas density are the two main factors affecting the RTS transmission behavior regardless of the size of the simulation box. Our MD results clearly indicate that in the working temperature range of the RTS from 100 K to 600 K, the higher the temperature and the lower the motor input rotation frequency, the higher the critical working helium density range allows.

## 1. Introduction

Nanomachines have attracted a lot of attention recently with the rapid development of nanotechnology [1,2] and the urgent requirements in chemical and biological engineering [3,4], especially after the 2016 Nobel Prize in Chemistry was issued to three scientists (i.e., Jean-Pierre Sauvage, Sir J. Fraser Stoddart, and Bernard L. Feringa) who have made brilliant contributions to the field [5]. The Nano-Electro-Mechanical System (NEMS) [6] is considered to be one of the important components in nanotechnology, which has the characteristics of quantum effect, interface effect, size effect, and so on. Carbon nanotubes (CNTs) show excellent mechanical [7,8], electrical [9,10], strain sensitive [11,12], and thermal [13,14] properties due to their special hexagonal structure, small size, low density, high strength-to-weight ratio, and high hardness [15]. Therefore, CNTs have become the most promising new type of nanomaterial in the construction of NEMS. In particular, CNTs have two excellent mechanical properties, one is the extremely high modulus and strength [16,17], and the other is the extremely low friction between adjacent layers of multi-walled carbon nanotubes (MWCNTs) [18,19]. With the two extraordinary mechanical properties, CNTs have found important potential applications for nano-devices such as nano-oscillator [20,21], nanobearing [22,23], and nanomotor [24,25]. The high modulus and high strength of the carbon material guarantees the stability of the carbon components working at gigahertz, and the extremely low friction ensures that the relative movement between adjacent components could be easily maintained. According to the characteristics of motion, the nanodevices can be separated into two groups. One is linear nanodevices, e.g., nano-oscillator, and the other is rotary nanodevices, e.g., nanomotor. Rotary nanomotors, as an important component in providing power for the movement of nanodevices, need to be designed according to the service environment, and its movement should be controllable [26]. Due to the lack of precise experimental equipment and limitation of experimental technology and other objective factors, it is difficult to conduct experimental research on nanotubes. Fortunately, as a mature simulation method, the molecular dynamics (MD) method [27,28] has become an important method for researchers to study the related properties of nanotubes, and provides a powerful tool that satisfies some of the requirements for designing the nanomotors at a nano scale.

In 2003, Fennimore et al. [24] observed the rotation behavior of CNTs through scanning electron microscopy in experiments for the first time. Since then, researchers have been using the rotation behavior of CNTs as driving components of nanodevices in the NEMS system. The driving force of rotary nanomotors could be provided by some external fields, such as light fields [29,30], electromagnetic fields [31,32], fluid fields [33,34], and thermal fields [23], etc. For instance, Barreiro et al. [23] investigated the transportation of cargoes at the sub-nanometer scale driven by temperature gradients along CNTs via experiments and MD simulations. Compared with the rotary nanomotors driven by the external fields, another type of rotary nanomotors [35,36] based on the MWCNTs without external field was subsequently proposed. The rotary nanomotors was in a gradientless temperature environment, the motion mechanism mainly relied on the inner tube, which loses geometric symmetry in the high temperature field to achieve the effect of rotation [35]. However, it was found that the rotation output signal has great randomness [23,24,29,30,31,32,33,34,35,36], and the uncontrollable output signal becomes a challenging problem that needs to be solved. Alternatively, Cai et al. [37,38] proposed a rotation transmission nano-system RTS consisting of an independent single-walled carbon nanotube (SWCNT) as a nanomotor and a group of coaxially arranged MWCNTs as nanobearing, in which the transmission of the rotation signal between the nanomotor and the nanobearing mainly depends on the interaction between adjacent ends. In addition, some researchers integrated nanomotor and nanobearing into another RTS model using a group of concentric MWCNTs [39,40,41,42,43]. Taking the advantage of the unique properties of boron nitride nanotube (BNNT) [44,45,46,47,48] and the excellent thermal and mechanical properties [49,50,51] of the heterogeneous CNT@BNNT, Zheng et al. [43] presented a novel RTS model of heterogeneous triple-walled nanotubes (TWNTs) based on CNTs and BNNT. The proposed RTS model was easier to control and the output signal was tunable in a wider temperature range, and the higher the system temperature, the higher the rotation transmission efficiency.

The rotary nanomotors and RTSs in the work mentioned above were studied in a vacuum environment. In fact, nanodevices may need to be manufactured or work in a specific medium environment. Shi et al. [52] explored the efficiency of CNT-based RTS in water solution, and they manipulated the transmission efficiency of RTS through the water-rotor interaction in the water box. Cai et al. [53] and Shi et al. [54] studied the rotation behavior of nanomotors and nanorings in argon environments, respectively. The results show that in a higher temperature environment, the nanomotors and the nanorings have a higher rotation frequency than that at a lower temperature. Research data over the past decade shows that among all inert gases, especially helium, it has no anesthetic “side effects” such as xenon, allowing this specific gas to be used in many clinical ischemia/reperfusion situations [55]. On the other hand, helium is usually adopted to be a shielding gas in experiments. The rotational behavior of carbon monoxide in the helium environment was studied via the experimental method by Surin et al. [56]. Meanwhile, in a research similar to [56], Gupta [57] showed that the formation of CNTs and fullerene in a pure carbon arc in helium atmosphere may involve graphene bubbles. Jost et al. [58] investigated the influence of the helium and nitrogen gas flow velocity and the laser pulse rate on the diameter distribution of SWCNTs. Hinkov et al. [59] also used helium and other gas as buffer gases to achieve the optical plasma temperature control during arc CNT growth. However, as far as we know, there is no report on the transmission characteristics of RTS in a helium environment.

The purpose of this work is to explore the transmission behavior of the RTS proposed by Zheng et al. [43] in a helium environment. When the RTS works in a helium environment, randomly moving helium atoms will attract or repel the atoms on the nanotubes, thereby generating fluid friction and affecting the transmission performance of the RTS. Clearly, it can be concluded from aforementioned studies [43,53,54] that the temperature and gas densities have a significant influence on the nano-system in a medium environment. Therefore, using the classical MD method, the influences of the system temperatures, helium densities and simulation box sizes on the transmission behavior of RTS in a helium environment are especially investigated, aiming at providing guidance for the potential applications of RTS in nanodevices.

## 2. Results and Discussion

In the RTS, the transmission behavior between the motor and the rotor mainly relies on the interaction between any two nanotube atoms, which includes interlayer van der Waals effects and end effects. The intratube interaction between atoms is a covalent bond, i.e., σ bond, while the intertube interaction is mainly due to the van der Waals force, i.e., π bond. The strength of the σ bond is much greater than that of the π bond. Around each intratube atom, except for the layer of atoms at both ends in the tube, there are three carbon atoms connected to it, which are connected by σ bonds, and each atomic bond is in a saturated state. However, there are only two atoms connected to the layer of atoms at the ends of the open-ended nanotubes, indicating that this layer of atomic bonds is not in an unsaturated state. After energy minimization, the rotor is in a state of equilibrium, and is then driven by the continuously rotating motor. Under the combining driving force of the interlayer van der Waals effects and the end effects between the tubes, the rotor starts to accelerate and rotate in the circumferential direction. The atom balance distance between the motor and the rotor is continuously destroyed due to the continuous rotation of the motor. At this time, the distance between the atoms in the equilibrium state increases and the atoms on the motor and the atoms on the rotor are attracted to each other under the combined action of the interlayer van der Waals effects and the end effects, leading the rotor to rotate in the circumferential direction due to the non-zero angular acceleration and oscillate in the axial direction. This relative movement also causes friction between the tubes. When the driving force and friction force reach a circumferential equilibrium, the rotor obtains a stable rotation frequency [43]. However, when the RTS works in a helium-filled zone, not only will there be friction between the motor and the rotor, but also the disordered movement of helium atoms will generate skin friction drag, which will affect the rotor’s motion behavior. Therefore, the rotor needs to completely overcome the friction between the tubes and the skin friction drag. If the driving force and the total friction reach a circumferential balance, the rotor can have a stable expected output speed.

During the simulation, the rotation behavior of the motor and rotor is described by its rotation frequency. In order to describe the relative rotation between the nanotubes, we define the ratio of rotation transmission (RRT) between the motor and the rotor as $\mathrm{RRT}=\frac{\omega_{r}}{\omega_{m}}$. When the value of RRT fluctuates around 1.0, the rotor is fully driven and will rotate synchronously with the motor. When the value is less than 1.0, the rotor is not fully driven, its output speed will be less than the motor input speed.

### 2.1. Comparison of Two Heterogeneous Rotation Transmission Nano-Systems

**Figure 1:**
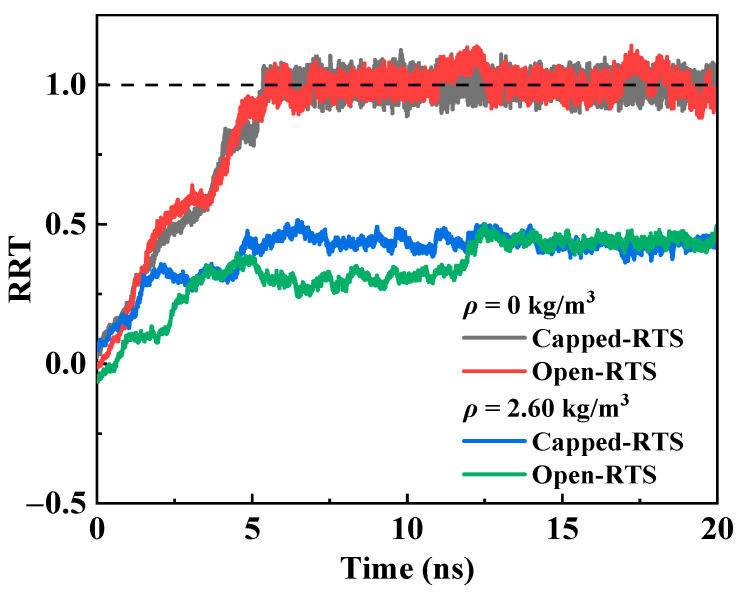
Time histories of RRT curves for the two RTS models with different rotor end structures in vacuum and helium environments.

Figure 1 shows the curves of RRT for two RTS models with different rotor end structures in vacuum and helium environments. In this section, the size of the simulation box is set to 8 nm × 8 nm × 8 nm, *ρ* is 2.60 kg/m^3^, the input *ω*_m_ is 200 GHz, and the system temperature is 300 K. It is found that when the RTS works in a vacuum environment, the RRT curves of the open-rotor RTS and the capped-rotor RTS almost coincide, and both can reach 1.0 within 5.5 ns. Therefore, the end structure of the rotor does not affect the transmission behavior of the RTS in the vacuum environment. However, when the RTS works in a helium environment with *ρ* = 2.60 kg/m^3^, the highest RRT of the open-rotor RTS and the capped-rotor RTS can only reach about 0.49, because of the increase in total frictional resistance caused by the action of helium atoms. Compared with the open-rotor RTS, the response time for the RRT of the capped-rotor RTS reaching 0.49 is shorter.

**Figure 2:**
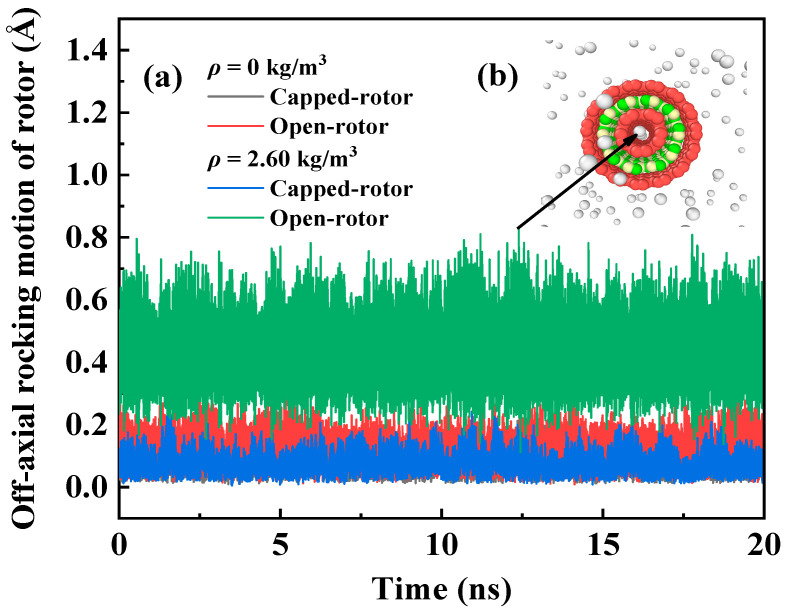
(**a**) Time histories of the off-axial rocking motion of the rotor with different end structures in vacuum and helium environments, and inset (**b**) atomic diagram of the open-RTS at a certain moment.

Regardless of whether the RTS works in a vacuum or helium environment, the motor and rotor will swing eccentrically during the movement, and the center of mass will deviate from the center line to a certain extent. This is one of the important factors that affect the stability of the RTS structure. The larger the off-axial rocking distance, the worse the stability of the RTS. Figure 2 depicts the time histories of the off-axial rocking motion of the rotor with different end structures in the vacuum and helium environment. It can be seen from Figure 2 that the degree of the off-axial rocking motion of rotor in a helium environment is greater than that in a vacuum environment due to the effect of helium atoms. The off-axial rocking distances of the open-rotor in the RTS working in vacuum and helium environment are about 0–0.38 Å and 0.2–0.8 Å, respectively, and those of the capped-rotor are about 0–0.2 Å and 0–0.25 Å, respectively. The off-axial rocking distances of the open-rotor are larger than those of the capped-rotor because helium atoms will run into the inner open-tube rotor when the RTS is surrounded by helium atoms, as shown in the inset of Figure 2b. The helium atoms in the open-rotor and the carbon atoms on the rotor are not in the equilibrium distance, which causes the helium atoms to attract or repel the inner tube, leading a larger off-axial rocking motion of rotor. However, the off-axis swing of the capped-rotor in the helium environment is almost negligible compared to that in the vacuum environment. Therefore, the capped-rotor RTS is more stable than the open-rotor RTS when they work in a helium environment. So capped-rotor RTSs will be adopted in the subsequent MD simulations.

### 2.2. Influence of Helium Density and Input Rotational Frequency of the Motor

**Figure 3:**
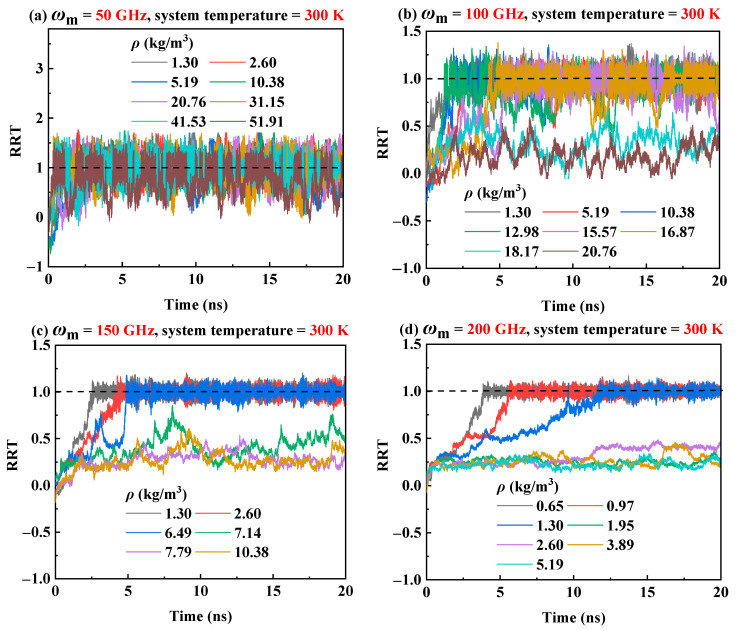
Time histories of RRT at 300 K with different helium densities and motor input rotation frequencies: (**a**) *ω*_m_ = 50 GHz, (**b**) *ω*_m_ = 100 GHz, (**c**) *ω*_m_ = 150 GHz, and (**d**) *ω*_m_ = 200 GHz.

To investigate the influence of helium density *ρ* and motor input frequency on the transmission performance of RTS, *ρ* is set from 0.65 kg/m^3^ to 51.91 kg/m^3^, and *ω*_m_ is set to 50 GHz, 100 GHz, 150 GHz, and 200 GHz, respectively. The system temperature is set to 300 K. Figure 3a–d plot the RRT curves in a helium environment with different gas densities in case of *ω*_m_ = 50 GHz, 100 GHz, 150 GHz, and 200 GHz, respectively. The results show that as *ρ* increases, meaning that more helium atoms enter the simulation box, the disordered movement of more helium atoms will generate greater skin friction drag, leading the transmission performance of RTS to become worse. It is manifested in two ways. One is that when *ρ* is smaller than a certain critical range, the RRT of RTS can reach 1.0, but when *ρ* is higher than this critical range, the RRT drops sharply. It is noted that the critical range relies on the input rotational frequency *ω*_m_ of the motor. It can be seen from Figure 3 that when *ω*_m_ is 50 GHz, 100 GHz, 150 GHz, and 200 GHz, the corresponding critical density range is 41.53–51.91 kg/m^3^, 15.57–16.87 kg/m^3^, 2.60–6.50 kg/m^3,^ and 0.97–1.30 kg/m^3^, respectively. When *ρ* exceeds the critical range, the skin friction drag of helium atoms to the RTS is increased. Although the rotor can be actuated by the motor, the driving force is not enough to completely overcome the total friction, resulting in the motor and rotor being unable to balance in the circumferential direction, so the RRT of the RTS is less than 1.0. Another way is that compared with the RRT curves of the RTS in a vacuum environment, the RRT curves fluctuate greatly when the RTS works in a helium environment with a gas density below the critical range, and they become disordered when the gas density is above the critical range.

**Figure 4:**
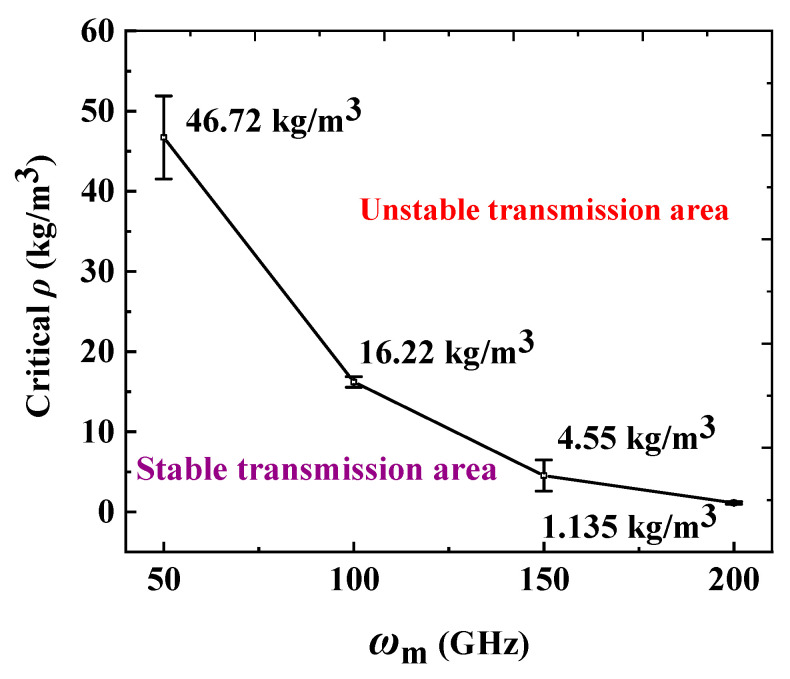
The schematic diagram of the stable and unstable transmission areas of the RTS.

According to the critical density range in Figure 3, Figure 4 gives the critical helium density error bar as a function of the input rotational frequencies of the motor. We respectively define the area under the curve and the area above the curve as stable and unstable areas. It can be seen from Figure 4 that as *ω*_m_ increases, the critical density range decreases, which suggests that the RTS can work stably in a helium environment with a high *ρ* when it works at a low motor rotation frequency, and can only work in a helium environment with a low *ρ* when the RTS works at a high motor rotation frequency.

### 2.3. Influence of the Size of Simulation Box

**Figure 5:**
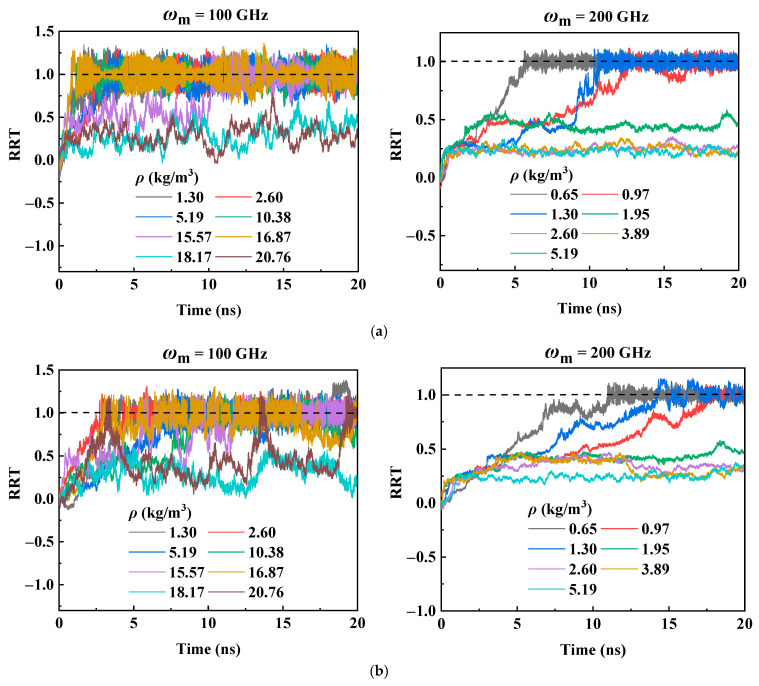
Time histories of the RRT curves for RTS under the cases of different simulation box sizes: (**a**) 16 nm × 16 nm × 8 nm, and (**b**) 32 nm × 32 nm × 8 nm.

In order to reflect more accurately the influence of *ρ* on the transmission performance of RTS, two sizes of the simulation box, i.e., 16 nm × 16 nm × 8 nm and 32 nm × 32 nm × 8 nm, are added for investigating the transmission performance of RTS in this section, in which the system temperature is 300 K and *ω*_m_ is set to 100 GHz and 200 GHz, respectively. For the same *ρ*, a larger simulation box has more helium atoms, as listed in Table 1 in Section 3.1. Figure 5 depicts the time histories of the RRT curves for RTS under the cases of different simulation box sizes. From Figure 5a,b, it can be found that when the size of the simulation box size is 16 nm × 16 nm × 8 nm or 32 nm × 32nm × 8 nm, the critical working *ρ* range of RTS at the corresponding *ω*_m_ of 100 GHz and 200 GHz are both 15.57–16.87 kg/m^3^ and 0.97–1.30 kg/m^3^, respectively, which are consistent with those obtained from the simulation box size of 8 nm × 8 nm × 8 nm (Figure 3b,d). Therefore, the simulation results are independent on the size of the simulation box.

### 2.4. Influence of System Temperature

**Figure 6:**
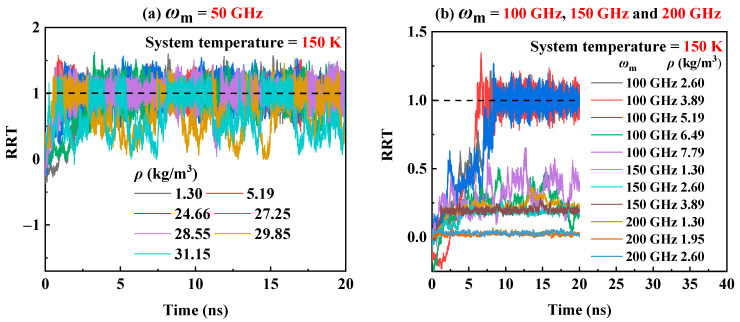
The curves of RRT at 150 K in a helium environment when (**a**) ω_m_ = 50 GHz, (**b**) ω_m_ = 100 GHz, 150 GHz, and 200 GHz.

**Figure 7:**
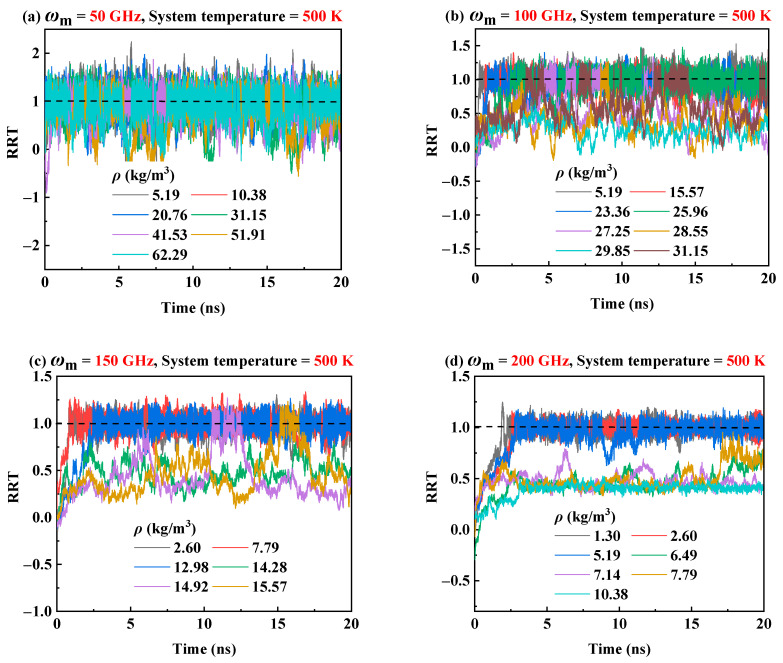
The curves of RRT at 500 K in helium environment when (**a**) *ω*_m_ = 50 GHz, (**b**) *ω*_m_ = 100 GHz, (**c**) *ω*_m_ = 150 GHz, and (**d**) *ω*_m_ = 200 GHz.

The transmission behavior of the RTS at the 300 K has been analyzed in Section 2.2. It is well known that temperature is one of the major factors that affect the transmission performance of the RTS [43,53,54]. Hence, in order to investigate the influence of system temperatures on the transmission performance of RTS in helium environment, three temperatures of 150 K, 300 K, and 500 K are considered. Figure 3 shows that when the system temperature is 300 K in case of *ω*_m_ = 50 GHz, 100 GHz, 150 GHz and 200 GHz, the corresponding critical working density range is 41.53–51.91 kg/m^3^, 15.57–16.87 kg/m^3^, 2.60–6.50 kg/m^3^, and 0.97–1.30 kg/m^3^, respectively. However, when the system temperature drops to 150 K, the critical working density range of the RTS will be reduced, as shown in Figure 6. It can be seen from Figure 6 that when *ω*_m_ is 50 GHz and 100 GHz, the RRT could reach 1.0 when *ρ* range is lower than 27.25–28.55 kg/m^3^ and 3.89–5.19 kg/m^3^ respectively. However, when *ω*_m_ is 150 GHz and 200 GHz, the transmission performance of RTS in the helium environment under the density range we studied is very poor. At higher temperatures, the thermal vibration of atoms/molecules in the system becomes more severe, and the nonbonding interaction at the adjacent ends of the rotor and the motor becomes more energetic. Therefore, when the system temperature rises within a certain range, the interlayer van der Waals effects and the end effects of RTS increase, so the driving force of the rotor increases with the increase of temperature [43]. The rotor rotational speed will accelerate as the environmental temperature rises [53,54]. When the system temperature rises to 500 K, although the skin friction drag of helium increases with the increase in temperature, a higher critical working density range of RTS is allowed because of the greater driving force with the increasing temperature. Figure 7 shows that when the system temperature is 500 K and *ω*_m_ is 50 GHz, 100 GHz, 150 GHz, and 200 GHz, the critical working gas density range is 51.91–62.29 kg/m^3^, 23.36–25.96 kg/m^3^, 7.79–12.98 kg/m^3^ and 2.6–5.19 kg/m^3^, respectively. Hence, the system temperature will seriously affect the critical working gas density range of the RTS.

**Figure 8:**
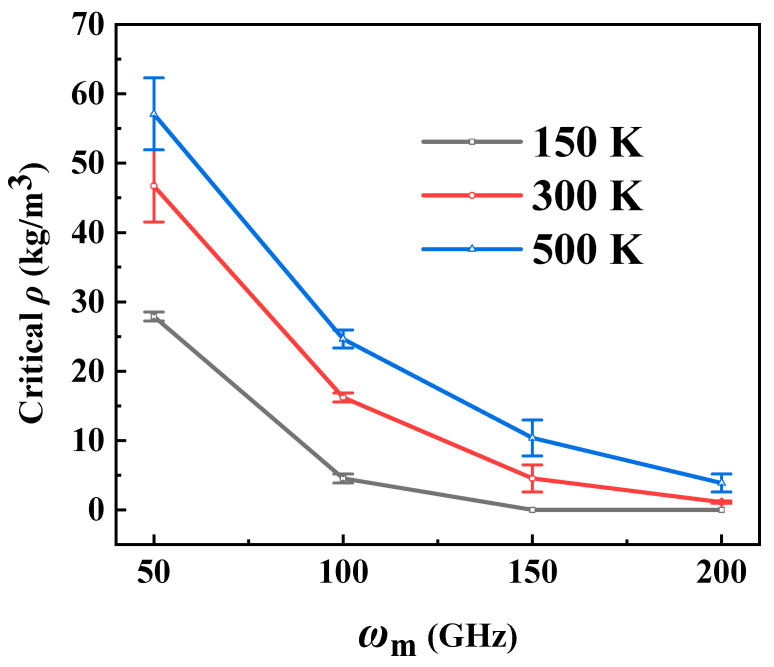
The relationship between the critical working helium density range and *ω*_m_ at different system temperatures.

In conjunction with Figure 3, Figure 6 and Figure 7, the influence curves of the system temperature and *ω*_m_ on the critical working density error bar are drawn in Figure 8. They clearly indicate that in the working temperature range of the RTS from 100 K to 600 K [43], the higher the temperature and the lower the motor input rotation frequency, the higher the critical working helium density range allows.

## 3. Models and Methods

### 3.1. RTS Model

**Figure 9:**
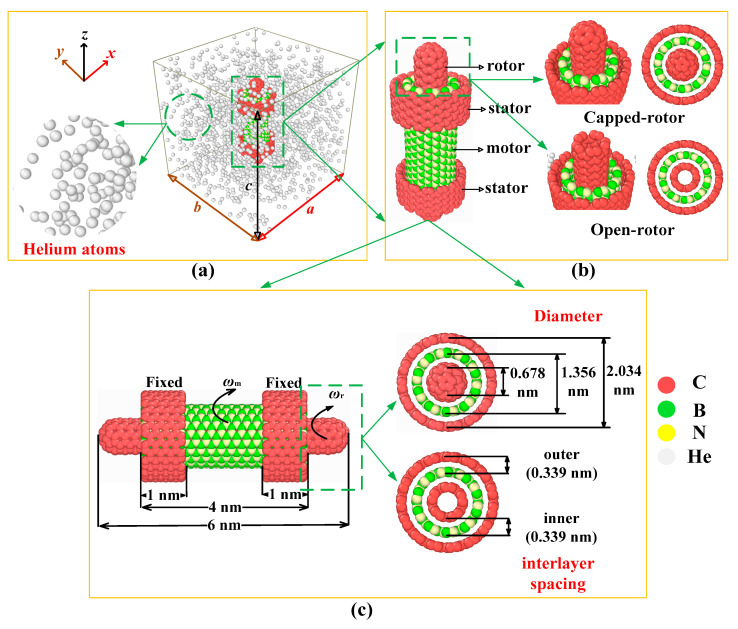
Rotation transmission nano-system (RTS) model made from CNT (5, 5)/BNNT (10, 10)/CNT (15, 15): (**a**) The RTS model in a helium environment, (**b**) the RTS model with different rotor end structures, and (**c**) the structure parameters of the RTS model.

In order to investigate the dynamic behavior of the RTS in a helium environment, a RTS made from CNT (5, 5)/BNNT (10, 10)/CNT (15, 15) in a helium-filled simulation box is set up, as shown in Figure 9. Figure 9a shows a heterogeneous TWNTs RTS made of CNTs and BNNT placed in the center of a simulation box with a boundary length of *a* × *b* × *c* in a helium environment. Figure 9b depicts two RTS models with different end structures of the rotor, one being the capped-rotor RTS model, and the other being the open-rotor RTS model. Figure 9c shows the construction parameters of the RTS model, where the inner CNT (5, 5) with a length of 6 nm and a diameter of 0.678 nm acts as rotor, the middle BNNT (10, 10) with a length 4 nm and a diameter of 1.356 nm acts as motor, and the two outer separated CNTs (15, 15) with a length of 1 nm and a diameter of 2.034 nm are fixed as stators. The inner and outer interlayer spacing are both set to 0.339 nm. The nanotubes of TWNTs in this model have a symmetrical layout along the *z* axis. The CNT stators are fixed during the MD simulations. *ω*_m_ and *ω*_r_ represent the rotation frequencies of motor and rotor, respectively. Table 1 lists the number of helium atoms corresponding to different helium densities *ρ* in the simulation box with different sizes.

### 3.2. MD Method

**Figure 10:**
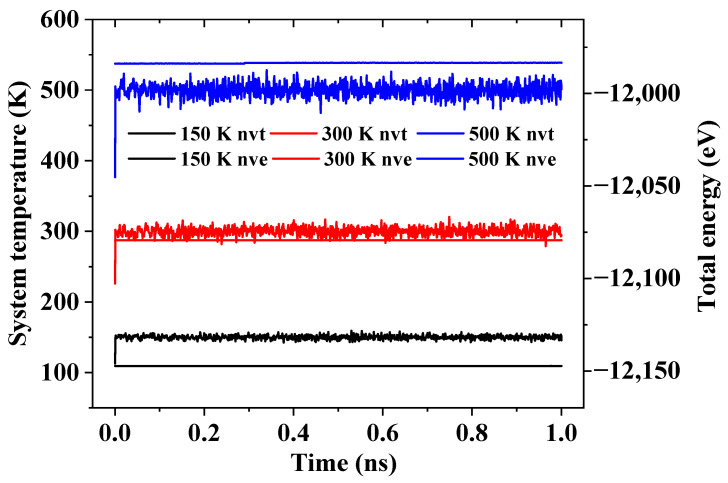
Convergence of system temperatures and system total energies during relaxation.

Initially, the helium atoms in the RTS model are laid out regularly in lines in the simulation box, and the boundaries for the global simulation box in each dimension are periodic. Consequently, at the beginning of the simulation, the structure is reshaped under minimized potential energy of the system by relaxing the system at the canonical NVT ensemble for 1 ns and the microcanonical NVE ensemble for 1 ns to obtain a reasonable layout of helium atoms with the RTS model, where N is the total number of particles in the system, V is the system’s volume, T is the absolute temperature, and E is the total energy in the system. As shown in Figure 10, it can be clearly seen that under a different system temperature, the total energy of the system is well converged during the relaxation. After the relaxation, the stators are fully fixed, and then the motor is applied with a continuous rotation at a given frequency through the command “fix move rotate”. This command essentially sets the angular velocity of each atom on the motor around the axis of rotation. The atoms on the rotor are free. The movement of rotor driven by the motor under the microcanonical NVT ensemble is recorded. For the systems of different system temperatures and different ω_m_, the time to reach equilibrium rotation frequency of the rotor is also different, and the specific range is also clarified by Zheng et al. [43]. In addition, when the speed of the motor increases, the time to reach equilibrium rotation frequency of the rotor also increases, so the total time for the simulation is 20 ns. The time step for all simulations is chosen to be 1 fs. LAMMPS [60] (Large-scale Atomic/Molecular Massively Parallel Simulator) is employed in the simulations.

The interaction among carbon atoms on CNTs is evaluated by the AIREBO potential [61,62]. The interaction among boron atom and nitrogen atom on BNNT is evaluated by the Tersoff potential [63]. The Lennard-Jones (L-J) 12-6 potential function is used to describe the interaction of the C, B, N, and He atoms [64,65,66]. The L-J interaction can be written as $U(r_{i}{}_{j})=4\varepsilon \left[{\left(\frac{\sigma }{r_{i}{}_{j}}\right)}^{12}-{\left(\frac{\sigma }{r_{i}{}_{j}}\right)}^{6}\right]$ ($r_{ij}$ ≤ $r_{c}$), where $r_{ij}$ represents the distance between the atoms, ε is the well-depth, σ is the size parameter, and $r_{c}$ is the cutoff distance. The Lorentz-Berthelot mixing rule [67,68] is used to determine the well-depth and size parameters between any two atom types. The parameters of the L-J 12-6 potential listed in Table 2. The cutoff distance of the L-J potential is 1.02 nm.

### 3.3. Simulation Strategies

To investigate the influence of different helium densities (*ρ*) on the transmission performance of RTS, the RTS is placed in the original simulation box with a size of 8 nm × 8 nm × 8 nm, and the *ρ* is set from 0.65 kg/m^3^ to 51.91 kg/m^3^. The volume of RTS accounts for 2.65% of the simulation box, and the effective working volume of helium accounts for 97.32% of the simulation box. The system temperature is set as 300 K.

To study the influence of system temperature on the transmission performance of RTS, three system temperatures, i.e., 150 K, 300 K, and 500 K are considered. The motor input rotation frequency (ω_m_) is set to 50 GHz, 100 GHz, 150 GHz and 200 GHz respectively.

To study the influence of the simulation box size on the RTS, the period boundary value is set to 4 times and 16 times of the original simulation box, i.e., the size of the simulation box is 16 nm × 16 nm × 8 nm and 32 nm × 32 nm × 8 nm, respectively. In the case of the same ρ, a larger simulation box has more helium atoms. The ω_m_ is set to 100 GHz and 200 GHz, and the system temperature is 300 K.

## 4. Conclusions

In this work, we have conducted MD simulations to study the transmission performance of the heterogeneous rotation transmission nano-system (RTS) based on carbon nanotubes (CNTs) and boron nitride nanotube (BNNT) in a helium environment. Considering the structural stability of the RTS model, we found that the capped-rotor RTS is more suitable for stable operation in a helium environment. Influences of some typical factors such as system temperature, helium density, size of simulation box, and input rotation frequency of motor were especially investigated. The MD simulation results showed that when the gas density is lower than a critical range, a stable output signal of the rotor can be obtained and the rotation transmission ratio (RRT) of RTS can reach 1.0, but as the gas density is higher than the critical range, the rotor cannot output stably due to the sharp drop of the RRT caused by the large friction between helium and the RTS. The results also showed that the system temperature and gas density are two main factors affecting the RTS transmission behavior regardless of the size of the simulation box, which clearly indicates that in the working temperature range of the RTS from 100 K to 600 K, the higher the temperature and the lower the motor input rotation frequency, the higher the critical working helium density range allows. The research in this work could provide theoretical input for the application of this type of nanodevices in a gas environment.

## Figures and Tables

**Table 1:** The number of helium atoms corresponding to different helium densities *ρ* in the simulation box with different sizes.

**8 nm × 8 nm × 8 nm**	***ρ* (kg/m^3^) **	0.65	0.97	1.30	1.95	2.60	3.89	5.19	6.49	7.14
		7.79	10.38	12.98	14.28	14.92	15.57	16.87	18.17	20.76
		23.36	24.66	25.96	27.25	28.55	29.85	31.15	41.53	51.91
		62.29								
	Number of atoms	50	75	100	150	200	300	400	500	550
		600	800	1000	1100	1150	1200	1300	1400	1600
		1800	1900	2000	2100	2200	2300	2400	3200	4000
		4800								
**16 nm × 16 nm × 8 nm**	***ρ* (kg/m^3^) **	0.65	0.97	1.30	1.95	2.60	3.89	5.19	10.38	15.57
		16.87	18.17	20.76						
	Number of atoms	200	300	400	600	800	1200	1600	3200	4800
		5200	5600	6400						
**32 nm × 32 nm × 8 nm**	***ρ* (kg/m^3^) **	0.65	0.97	1.30	1.95	2.60	3.89	5.19	10.38	15.57
		16.87	18.17	20.76						
	Number of atoms	800	1200	1600	2400	3200	4800	6400	12,800	19,200
		20,800	22,400	25,600						

**Table 2:** Lennard-Jones parameters for the interatomic van der Waals interactions.

Atom *i*-*j*	*ε* (eV)	*σ* (nm)
C-C	0.00373	0.3400
B-B	0.00411	0.3453
N-N	0.00628	0.3365
He-He	0.00219	0.2559
C-B	0.00391	0.3427
C-N	0.00484	0.3383
C-He	0.00285	0.2980
B-He	0.00300	0.3006
N-He	0.00370	0.2962

## Sample Availability

Samples of the compounds are not available from the authors.
